# Evidence in Favor of an Alternative Glucocorticoid Synthesis Pathway During Acute Experimental Chagas Disease

**DOI:** 10.3389/fendo.2019.00866

**Published:** 2020-01-08

**Authors:** Esdras da Silva Oliveira Barbosa, Eduardo A. Roggero, Florencia B. González, Rocío del Valle Fernández, Vinicius Frias Carvalho, Oscar A. Bottasso, Ana R. Pérez, Silvina R. Villar

**Affiliations:** ^1^Institute of Clinical and Experimental Immunology of Rosario (IDICER-CONICET-UNR), Rosario, Argentina; ^2^Laboratory of Inflammation, Oswaldo Cruz Institute, Oswaldo Cruz Foundation, Rio de Janeiro, Brazil; ^3^National Institute of Science and Technology on Neuroimmunomodulation (INCT-NIM), Rio de Janeiro, Brazil; ^4^Center for Research and Production of Biological Reagents (CIPREB), Faculty of Medical Sciences, National University of Rosario, Rosario, Argentina

**Keywords:** adrenal glands, glucocorticoid, ACTH, *Trypanosoma cruzi*, EPAC2, IL-1β, PGE2, ACTH-independent

## Abstract

It is well-established that infectious stress activates the hypothalamus–pituitary–adrenal axis leading to the production of pituitary adrenocorticotropin (ACTH) and adrenal glucocorticoids (GCs). Usually, GC synthesis is mediated by protein kinase A (PKA) signaling pathway triggered by ACTH. We previously demonstrated that acute murine Chagas disease courses with a marked increase of GC, with some data suggesting that GC synthesis may be ACTH-dissociated in the late phase of this parasitic infection. Alternative pathways of GC synthesis have been reported in sepsis or mental diseases, in which interleukin (IL)-1β, prostaglandin E2 (PGE2), and/or cAMP-activated guanine nucleotide exchange factor 2 (EPAC2) are likely to play a role in this regard. Accordingly, we have searched for the existence of an ACTH-independent pathway in an experimental model of a major parasitic disease like Chagas disease, in addition to characterizing potential alternative pathways of GC synthesis. To this end, C57BL/6 male mice were infected with *T. cruzi* (Tc), and evaluated throughout the acute phase for several parameters, including the kinetic of GC and ACTH release, the adrenal level of MC2R (ACTH receptor) expression, the p-PKA/PKA ratio as ACTH-dependent mechanism of signal transduction, as well as adrenal expression of IL-1β and its receptor, EPAC2 and PGE2 synthase. Our results reveal the existence of two phases involved in GC synthesis during Tc infection in mice, an initial one dealing with the well-known ACTH-dependent pathway, followed by a further ACTH-hyporesponsive phase. Furthermore, inflamed adrenal microenvironment may tune the production of intracellular mediators that also operate upon GC synthesis, like PGE2 synthase and EPAC2, as emerging driving forces for GC production in the advanced course of Tc infection. In essence, GC production seems to be associated with a biphasic action of PGE2, suggesting that the effect of PGE2/cAMP in the ACTH-independent second phase may be mediated by EPAC2.

## Introduction

The hypothalamus–pituitary–adrenal (HPA) axis is activated in diverse stressful situations, like pathological and metabolic disorders (1) or infectious diseases (2, 3), to preserve homeostasis (4) by controlling the availability of glucocorticoid (GC) hormones: corticosterone (CT) in rodents and cortisol in humans (4–6). Adrenocorticotropic hormone (ACTH) is the main stimulus for GC synthesis and release, acting through the melanocortin 2 receptor (MC2R). MC2R activation induces the synthesis of the second messenger cAMP, which, in a protein kinase A (PKA)-dependent fashion, induces the expression of many steroidogenic enzymes transforming cholesterol to GC (7).

Since pituitary disorders lead to secondary adrenal insufficiency (8), elevated GC concentrations have been traditionally ascribed as being due to a pituitary-stimulated increase of ACTH. Nevertheless, in the last decades, it became evident that alternative pathways of GC steroidogenesis may also occur in the context of some pathological situations. Patients with sepsis (9–12), or undergoing surgery (13, 14), as well as presenting malignant diseases or depression (15) often show in plasma-augmented GC amounts without changes in ACTH levels (16, 17). The dissociation between the ACTH and GC levels during critical illnesses may be envisioned as an adaptive phenomenon addressed to preserve elevated GC levels to respond as appropriately as possible to the stress-related needs. One possible alternative pathway involves the production of cAMP (in an ACTH-independent fashion), and the so-called cAMP-activated guanine nucleotide exchange factor 2 (EPAC2) (18–21), whose positive effects upon the steroideogenic pathway are exerted through mechanisms not yet fully described. Diverse mediators may be involved in the cAMP rise in the absence of ACTH, like prostaglandins (22, 23), and indirectly, some inflammatory cytokines (24, 25).

*Trypanosoma cruzi* (Tc) is a protozoan parasite causing Chagas disease, a main parasitic disease in Latin America. Chagas disease is currently spreading in a non-vector way throughout the world due to migratory flows. The parasite usually elicits an intense systemic response able to damage essential organs, i.e., heart and digestive tract (26, 27), causing disability. Moreover, oral breaks course with high lethality (28, 29). We previously demonstrated that Tc acute infection in C57BL/6 mice induces a strong release of GC, which is critical to mice survival (30, 31). Further studies developed in Tc-infected mice suggested that an ACTH-GC dissociation phenomenon may also occur in this protozoan infection. In fact, findings recorded from a single time point along the course of the acute infection showed that higher circulating levels of GC coexisted with slight ACTH amounts (32, 33), raising the view of a GC-driven negative feedback as playing a role in this regard.

Given this background, we searched for the occurrence of an ACTH-independent pathway in an experimental model of acute Chagas disease in addition to characterizing potential alternative pathways of GCs synthesis. Here, we evaluated throughout infection the kinetics of ACTH and GC production and intracellular pathways involved in GC synthesis in the adrenal gland. To discriminate ACTH-dependent from -independent pathways, Tc-infected mice were also assessed for MC2R expression and the PKA-pathway activation as a correlate of the ACTH-pathway activation, with the adrenal expression of interleukin (IL)-1β and its receptor (IL-1R), prostaglandin E2 (PGE2) synthase, and EPAC2 being studied as factors involved in the ACTH-independent pathway.

## Materials and Methods

### Mice and Experimental Infection

C57BL/6 male mice, aged 6–8 weeks, were obtained from the Animal Facilities of Faculty of Medical Sciences, National University of Rosario (FCM-UNR). Trypomastigotes of the Tulahuen strain of Tc, corresponding to Tc lineage VI (34) were used. Mice were infected with 200 viable trypomastigotes subcutaneously. Parasitemia and the survival time were recorded following infection, to monitor the systemic repercussion of the acute disease, as previously reported (32).

### Plasma ACTH and CT

Assessment of basal and infection-induced hormones was performed as previously reported (30, 32). Mice were housed individually 1 week before the beginning of the experiments and kept single-caged throughout the infection in temperature, and light-controlled rooms (light cycle from 7:00 a.m. to 7:00 p.m.). Plasma samples for hormone measurements were obtained from the tip of the tail between 8:00 and 10:00 a.m. (30, 32). Following that, blood was taken by cardiac puncture and adrenal glands were removed for other approaches detailed below. Plasma CT (IBL International, Hamburg, Germany) and ACTH levels (MD Bioproducts, Zurich, Switzerland) were determined by ELISA.

### Plasma and Intra-adrenal Cytokine Measurements

Plasma and adrenal glands were obtained from control and Tc-infected animals throughout acute infection. Plasma IL-1β was measured by specific two-site enzyme-linked immunosorbent assay (ELISA) using an ELISA kit according to the manufacturer's specifications (Pharmingen, USA). Plasma TNF-α, IFN-γ, and IL-6 were measured using a murine BD Cytometric Bead Array (BD Biosciences, USA). Intra-adrenal IL-1β mRNA levels were assessed by RT-qPCR, as below described. All samples were assayed in duplicate.

### Immunoblot Assays

Adrenal glands were homogenized in 4 volumes of 300 mmol/L sucrose with 1× protease inhibitor cocktail and 1× phosphatase inhibitor cocktail (SIGMA, Saint Louis, USA). Homogenates were centrifuged at 1,000*g* to remove unbroken cells, nuclei, and heavy membranes, based on previous studies (35). Proteins were quantified according to Lowry technique (36). For protein detection, samples were subjected to sodium dodecyl sulfate–polyacrylamide gel electrophoresis (SDS-PAGE) and electroblotted onto polyvinyl difluoride (PVDF) membranes (PerkinElmer Life Sciences, Inc., Boston, MA). Membranes were incubated with primary anti-mouse antibodies (anti-IL-1R, anti-PKA, anti-p-PKA, anti-EPAC2, anti-GAPDH, and anti-PGE2 synthase from Santa Cruz Biotechnology). The expression of total and phosphorylated isoforms of both PKA was analyzed by stripping in the same membrane. Finally, protein levels were detected by an enhanced chemiluminescence detection system (Pierce ECL, Thermo Fisher Scientific, USA). Immunoreactive bands were quantified by densitometry using the Image J software (imagej.nih.gov).

### Immunohistochemical Staining

Immunohistochemistry studies were performed on 4-μm paraffin sections from adrenal glands. Sections were deparaffinized with xylene, rehydrated in a gradient series of alcohol (100, 95, and 45% alcohol) and rinsed in PBS. Each section was covered with 0.3% peroxyacetic acid for 15 min to block endogenous peroxidase activity and microwaved for antigen retrieval (100 W, 5 min × 3 min), and cooled at room temperature (RT) for 20 min. Then, sections were incubated with the anti-MC2R (Santa Cruz Biotechnology, dilution 1/50) at RT during 60 min, and then rinsed again. This step was followed by incubation with a streptavidin–biotin–peroxidase antibody complex (BD Pharmingen) for 30 min at RT. Slides were then treated with streptavidin peroxidase reagent for 10 min. The sections were visualized with 3,3′-diaminobenzidine (DAB), counterstained with hematoxylin, and mounted in mounting medium for microscopical observation.

### RNA Isolation, cDNA Synthesis, and qPCR

Total RNA was isolated from adrenals using TRI Reagent (Genbiotech). cDNA was synthesized from 2 μg of total RNA by extension of oligo dT primers (Invitrogen, Carlsbad, CA, USA) with M-MuLV reverse transcriptase (Fermentas, Vilnius, Lithuania) according to the manufacturer's instructions. qPCR using 5X HOT FIREPol® Eva Green qPCR Mix Plus (Solis BioDyne, Tartu, Estonia) was performed in a StepOne Plus Real-Time PCR System (Thermo Fisher Scientific). Thermal cycling conditions were 15 min at 95°C followed by 40 PCR cycles of denaturing at 95°C for 15 s, 25 s for annealing at 60°C, and 25 s for elongation at 72°C. Fluorescence readings were performed during 10 s at 80°C before each elongation step. RPL13a (Gene ID: 22121) transcript was also measured and used as endogenous control to normalize the expression of mRNA determinations. External curves constituted by serial dilutions of cDNA of the transcript to be quantified were included in each run. Data are expressed as fold change with respect to RPL13a. Primer sequences are detailed in Table 1.

**Table 1:** Primer sequences and expected amplification products.

**Transcript**	**Forward primers**	**Reverse primers**	**Product size (bp)**
*RPL13a*Rpl13a, Gene ID: *22121*	*RPL13a-F* 5′-gca tga ggt cgg gtg gaa g-3′	*RPL13a-R*5′-ctc cac att ctt ttc tgc ctg ttt-3′	133
*IL-1r1*Il1r1, Gene ID: *16177*	*IL-1r1-F* 5′-tac agg gac tcc tgc tct ggt t-3′	*IL-1r1-R*5′-ccc tcc aag acc tca ggc aa-3′	152
*IL-1β*Il1b, Gene ID: *16176*	*IL-1β-F* 5′-agc tga aag ctc tcc acc tca at-3′	*IL-1β-R*5′-gtg ggt gtg ccg tct ttc att a-3′	163
*EPAC2*Rapgef4, Gene ID: *56508*	*EPAC2-F* 5′-gta cta cag gag cca gcc ctt-3′	*EPAC2-R*5′-atg gcc ttc gag gct cta atc t-3′	149
*Ptgs2*Ptgs2, Gene ID: *19225*	*Ptgs2-F* 5′-agt tca tcc ctg acc ccc aag-3′	*Ptgs2-R*5′-gaa aag gcg cag ttt atg ttg tct-3′	185
*Mc2r*Mc2r, Gene ID: 17200	*Mc2r-F* 5′-gac ctt ctg ccc aaa taa ccc tt-3′	*Mc2r -R*5′-cgg ttg cag aag agc atc ctt t-3′	159

*Specific selected primers for IL-1β, IL-1r1, EPAC2, Ptgs2, Mc2r, and RPL13a transcripts (quantitative polymerase chain reaction). RPL13a, ribosomal protein L13A; IL-1r1, interleukin 1 receptor type I; IL-1β, interleukin 1 beta; EPAC2, rap guanine nucleotide exchange factor (GEF) 4; Ptgs2, prostaglandin-endoperoxide synthase 2; Mcr2, melanocortin 2 receptor; bp, base pair*.

### Statistics

Data are shown as mean ± standard error of the mean (SEM), unless otherwise stated. Statistical analysis was performed by the non-parametric analysis of variance Kruskal–Wallis followed by Dunn post-test (*k* > 2) or *U* de Mann–Whitney test (*k* = 2). The GraphPad Instat 4.0 software (GraphPad, California, USA) was used for statistical analyses, and differences were considered significant when *p* value was <0.05.

## Results

### Tc Infection Induces Both ACTH-Dependent and -Independent Phases of GC Secretion

In Tc-infected C57BL/6 mice, parasitemia begins to be evident from day 7 post-infection (pi), followed by a marked and progressive increase (data not shown). As seen in earlier studies, infection was lethal in all animals with a mean survival time of 24–26 days (31, 32).

**Figure 1:**
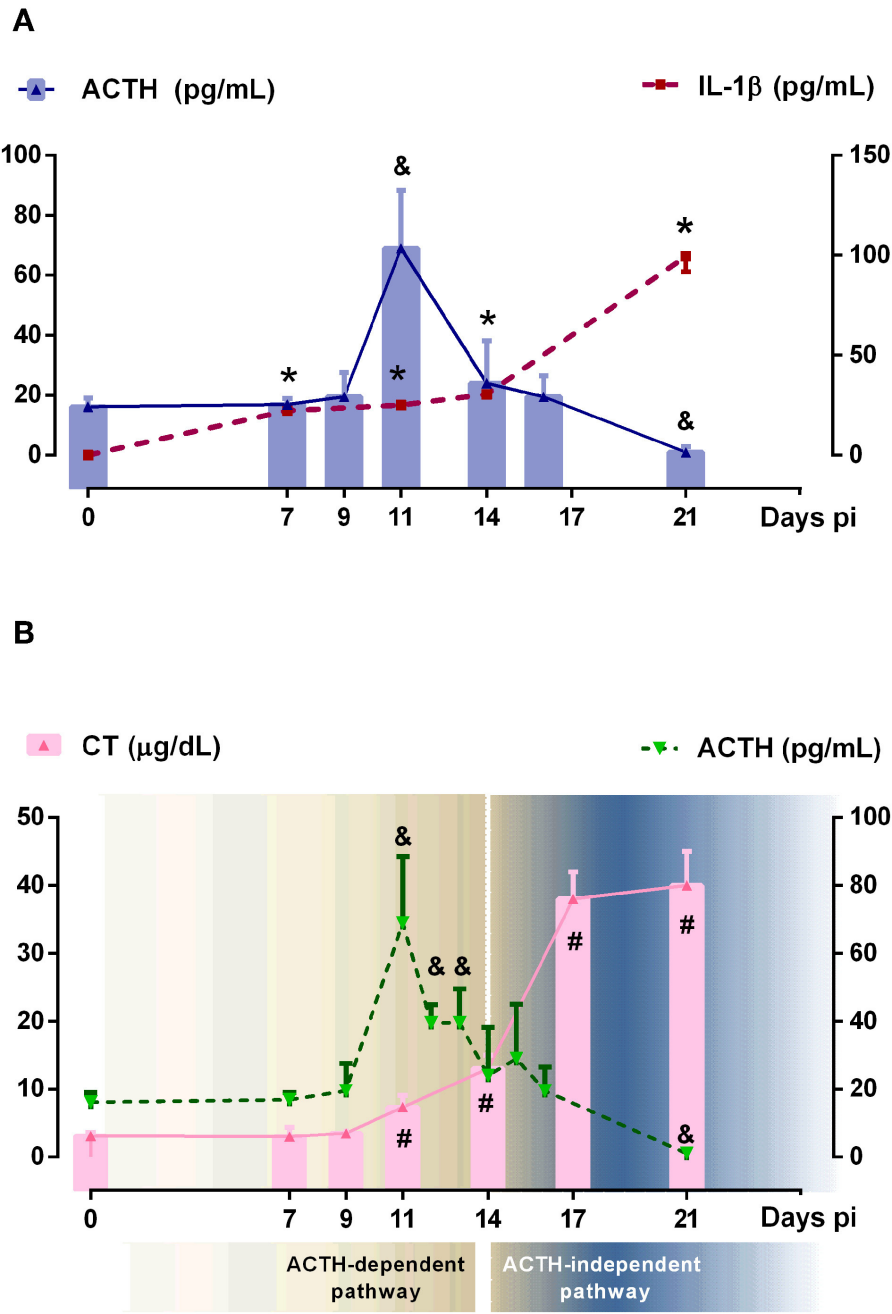
Relationship between ACTH, CT and IL-1β systemic levels. **(A)** IL-1β and ACTH showed an independent behavior after 14 days pi. Solid bars represent mean serum ACTH levels and dashed lines indicated serum IL-1β concentrations [ACTH: ^&^*p* < 0.05 vs. day 0 pi; IL-1β: *p < 0.05 vs. day 0 pi]. **(B)** ACTH and CT showed the classical coupled pattern of secretion until day 14 pi (defined as ACTH-dependent pathway) and a dissociated behavior afterwards (defined as ACTH-independent pathway) Solid bars correspond to CT levels and dashed line represents ACTH levels [ACTH: ^&^*p* < 0.05 vs. day 0 pi; CT: ^#^*p* < 0.05 vs. day 0 pi]. In all cases, lines are interpolations of mean values, thus approximating the time course of parameter levels between the measured time points. pi, post-infection.

To investigate the dynamic of ACTH and GC secretion in Tc-infected mice, plasma samples were obtained at different time points following infection. The main GC hormone present in rodents is CT and in humans cortisol. Basal CT levels in blood from non-infected mice were 2.1 ± 1.3 μg/dl, while basal ACTH concentrations were 16.2 ± 2.8. GC rise began to be observed from day 7 pi and increased progressively until day 21 pi (20-fold increase), while ACTH peaked by day 11 pi (4.3-fold increase), further lowering to values seen in control mice at day 14 pi, to reach quite reduced amounts by day 21 pi. These results showed that GC secretion is only matched to ACTH levels nearly during the first 2 weeks of infection, being ACTH-uncoupled afterwards (Figure 1A).

Tc infection increased plasma levels of IL-1β as well as other HPA axis-activating cytokines such as TNF-α, IFN-γ, or IL-6 (Supplementary Figure 1A). Among pro-inflammatory cytokines involved in the HPA axis activation, IL-1β is the most potent one. As can be seen in Figure 1A, the rise of IL-1β was observed at day 7 pi, probably constituting the main stimulus for ACTH-triggered CT synthesis in the initial response, whereas in more advanced infection, ACTH release seems not to be fueled by IL-1β (the same was true for TNF-α, IFN-γ, and IL-6). It follows that, during Tc infection in C57BL/6 mice, GC secretion does exhibit a dual control: an initial ACTH-dependent mechanism followed by an ACTH-uncoupled one (Figure 1B).

### ACTH-Dependent Functional Response Is Evidenced by the P-PKA/PKA Ratio

**Figure 2:**
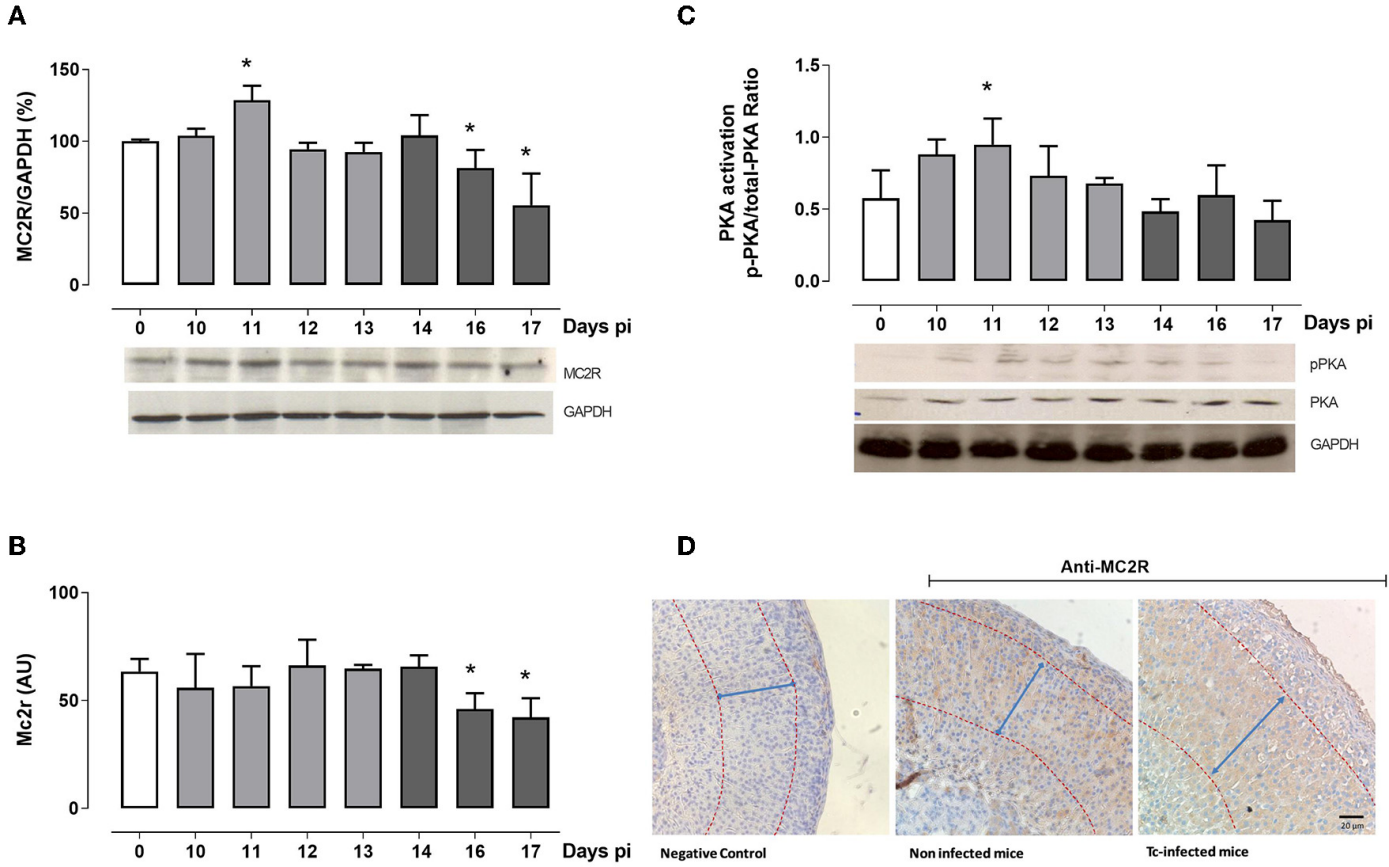
ACTH-dependent pathway analysis. **(A)** Western blot analyses of the MC2R expression throughout infection. Bars represent the densitometry with data from day 0 pi taken as 100%. Optical density was normalized to GAPDH. In the representative blot, lines are numbered according to the day pi. **(B)** MC2R mRNA levels in adrenal cells from Tc-infected mice at different days pi. **(C)** Western blot analyses of the p-PKA/PKA ratio at different days pi. In the representative blot, lines are numbered according to the day pi. Basal levels are represented by a white column; light gray columns represent p-PKA/PKA ratio levels during the ACTH-dependent phase; and dark gray columns represent p-PKA/PKA ratio levels during the ACTH-independent phase. **(D)** Immunohistochemical localization of MC2R in the adrenal cortex (magnification 20×). Positive immunoreactivity was observed in the fascicular zone from both non-infected (middle panel) and Tc-infected mice (14-day pi; right panel). Left panel shows the negative control. Tc-infected animals evidenced a clear hyperplasia of the *zona fasciculata* (demarcated with arrows). Results are expressed as mean ± SEM, from 3 to 5 mice/group/day. A representative experiment from three independent series is shown. *p < 0.05 vs. day 0 pi. AU, arbitrary units; Tc, *Trypanosoma cruzi*; pi, post-infection.

ACTH stimulates GC production through MC2R and also regulates MC2R gene and protein expression. The ligation of ACTH to MC2R activates the adenylyl cyclase cascade, leading to cAMP production. This step is followed by phosphorylation of cAMP-dependent PKA and the subsequent activation of several transcription factors inducing the expression of steroidogenic enzymes, like StAR (Supplementary Figure 1B). The latter in fact occurred during Tc. As seen in Figure 2A, MC2R protein expression peaked at day 11 pi, matching with higher ACTH levels. In the following days, MCR2 expression decreased, coinciding with the lowest ACTH plasma concentration. Consistent with protein data, MC2R gene expression decreased after 16 days pi (Figure 2B). In line with higher MCR2 protein expression, the p-PKA/PKA ratio revealed its highest point at day 11 pi (Figure 2C). Moreover, we also verify that MC2R expression was restricted to the zona fasciculata in Tc-infected animals (Figure 2D). The poor signaling shown by the MC2R/PKA pathway after 14 days pi indicates that mediators other than ACTH sustain the late GC synthesis in Tc-infected animals, reinforcing the view that GC synthesis is ACTH-dependent only in the first period of infection, and further becomes ACTH-independent.

### IL-1β and IL-1RI Are Expressed in Adrenal Glands During Infection

**Figure 3:**
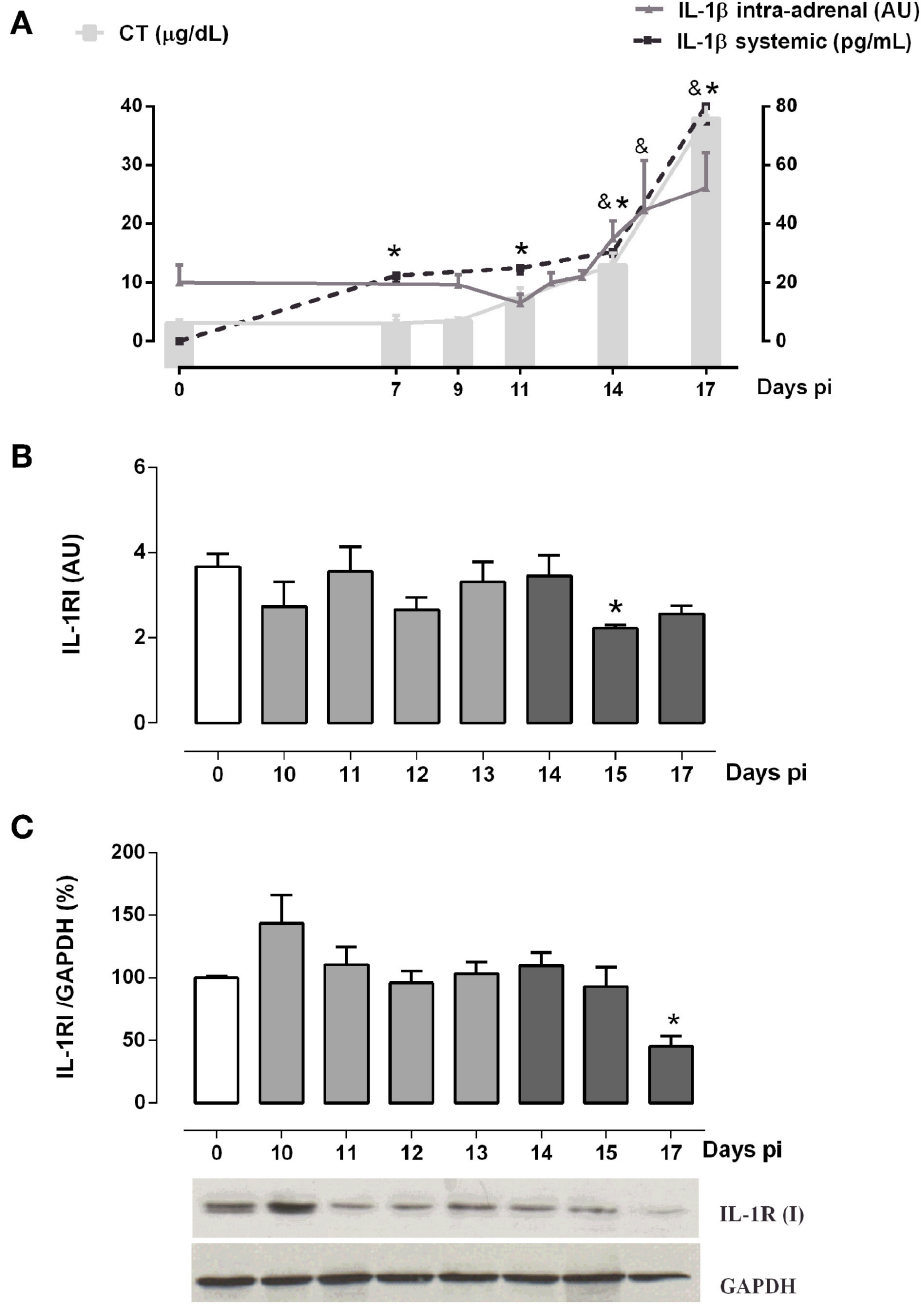
Intra-adrenal expression of IL-1β and IL-1RI in Tc-infected mice. **(A)** IL-1β and CT exhibited similar secretion kinetics throughout infection. Mean serum levels of CT are indicated by solid bars, while dashed lines illustrate IL-1β levels [IL-1β systemic: *p < 0.05 vs. day 0 pi; IL-1β intra-adrenal: ^&^ *p* < 0.05 vs. day 0 pi]. **(B)** IL-1RI mRNA expression was diminished at day 15 pi. **(C)** Western blot analyses of IL-1RI expression throughout infection showed a down-regulation at day 17 pi. Basal levels are represented by a white column; light gray and dark gray columns represent IL-1β transcription levels during the ACTH-dependent phase and ACTH-independent phases, respectively. Results are expressed as mean ± SEM, from 3 to 5 mice/group/day. IL-1β and IL-1RI transcripts were normalized against RPL13a gene (Gene ID: 22121) as endogenous control. Data correspond to a representative experiment from three independent rounds. *p < 0.05 vs. basal expression (day 0 pi). AU, arbitrary units; pi, post-infection.

Besides the hypothalamic effects of IL-1β, some studies showed that it also stimulates the steroidogenesis *in vitro* (37, 38), whereas human adrenal cells are also able to produce IL-1β (25). Since intra-adrenal production of IL-1β may represent an autocrine/paracrine factor involved in GC synthesis during the ACTH-independent phase, adrenal glands from Tc-infected mice were next studied for the expression of IL-1β and their receptor. In Tc-infected mice, the increase in GC levels not only occurred in parallel with the systemic elevation of IL-1β, but also with an increase in the IL-1β synthesis within adrenal glands (Figure 3A). Adrenal IL-1β transcripts begin to increase after 14 days pi, coinciding with the onset of the ACTH-independent phase.

Since IL-1β may locally signal through its receptor to enhance GC secretion in the ACTH-independent phase, the intra-adrenal expression of IL-1R1 was also investigated (Figures 3B,C). IL-1RI mRNA contents paralleled protein counterparts, showing no gross changes throughout infection, except on days 17 and 15 pi where both levels are, respectively, diminished (Figures 3B,C). These results suggest that IL-1β/IL-1RI signaling was off in the ACTH-independent phase.

### EPAC2 and PGE2 Synthase Cell Signaling-Related Factors Are Linked to GC Synthesis During the ACTH-Independent Phase

**Figure 4:**
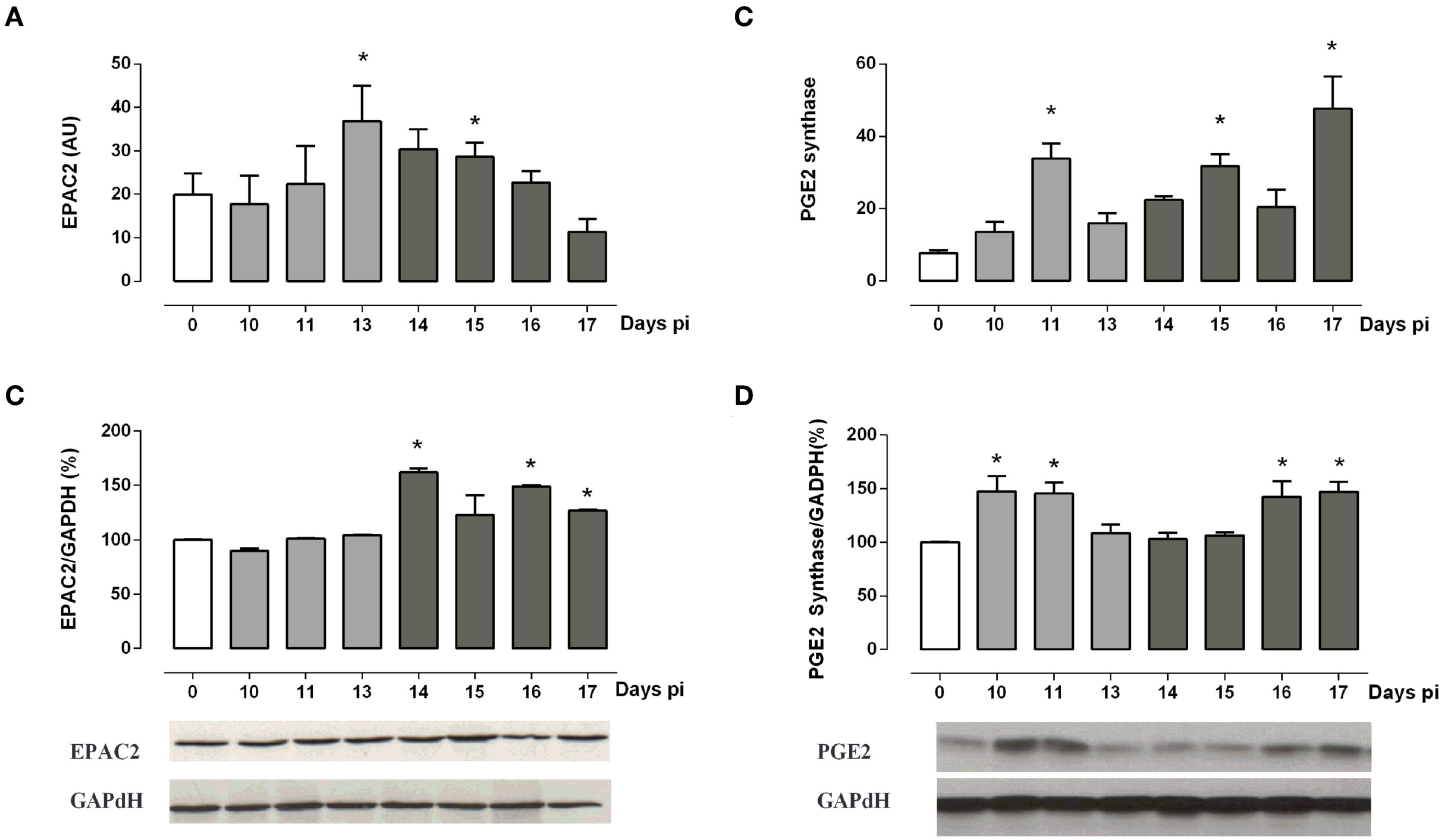
ACTH-independent pathway analysis. **(A,B)** EPAC2 mRNA and protein expression are increased during the ACTH-independent phase. **(C,D)** PGE2 synthase mRNA and protein are expressed throughout infection, showing a peak during both the ACTH-dependent and independent phases. Bars from immunoblots represent the densitometry with data from day 0 pi taken as 100%. Optical density was normalized to GAPDH. Basal levels are represented by a white column; light gray and dark gray columns represent IL-1β transcription levels during the ACTH-dependent phase and ACTH-independent phases, respectively. EPAC2 and PGE2 synthase transcripts were normalized against RPL13a gene (Gene ID: 22121) as endogenous control. Results are expressed as mean ± SEM, from 3 to 5 mice/group/day. A representative experiment from three independent series is shown. *p < 0.05 vs. basal expression (day 0 pi). AU, arbitrary units; pi, post-infection.

Besides PKA, EPAC2 may be involved in GC synthesis. Aimed at evaluating whether EPAC2 may play a role in the ACTH-independent phase, we next assessed EPAC2 expression. As depicted in Figure 4, EPAC2 mRNA reached high levels between 13 and 15 days pi (Figure 4A), whereas its protein content attained elevated concentrations after 14 days pi (Figure 4B), pointing out that EPAC2 is likely to be involved in the alternative pathway for steroidogenesis from Tc-infected mice.

Additionally, in the ACTH-independent phase, the cAMP supply may be sustained by PGE2. Measurements of PGE2 synthase mRNA and its protein showed two peaks of expression (Figures 4C,D). The first one coincides with the maximum release of ACTH, while the second weave is evident from 15 to 17 days pi, paralleling the ACTH-independent phase.

Overall, present data support the view that PGE2 may stimulate the adrenal cAMP production during both the ACTH-dependent and -independent phases, likely exerting a positive regulatory role on GC production via EPAC2 during the ACTH-independent phase.

## Discussion

The HPA axis is a dynamic system regulating the synthesis and release of adrenal GC during stressful conditions. Particularly, during states of immune hyperactivity, a rapid increase of GC is critical to mount an efficient anti-inflammatory response and hence preserving the energy supply required by immune cells. In the context of acute experimental Chagas disease, the relevance of HPA activation and the consequent role of GC as endogenous anti-inflammatory agents are undoubtful (30, 31). The fact that GC rise coincided with the highest circulating amounts of ACTH, together with an intensified adrenal MC2R expression and an enhanced p-PKA/PKA ratio, corroborate the existence of an ACTH-dependent pathway of GC synthesis at the early stage of Tc infection. Furthermore, these findings match with our earlier observations showing an evident adrenal hyperplasia accompanied by an enhanced steroidogenic machinery since StAR, CYP11A1, CYP11B1, and 11β-HSD1 expression are increased in this period (32).

Expanding our former results (32), we now reveal that infection-driven GC rise is coupled to ACTH solely during the first 2 weeks, to further become dissociated. The uncoupled ACTH-GC response observed in the second phase of infection denote the existence of ACTH-independent mechanisms maintaining the supply of GC. The occurrence of changes in the adrenal microenvironment conditioned by the infection may be central for such ACTH-independent GC secretion.

For instance, constitutive activation of MC2R or their signaling molecules has been thought as likely accounting for GC production in an ACTH-independent form (39, 40). However, this scenario does not occur in the late phase of Tc infection, since the MC2R/PKA pathway was evidently downregulated just after 2 weeks, favoring the lack of response to ACTH even in the presence of hormone basal levels. *In vitro* studies in 24-h LPS-exposed adrenal cells revealed a reduced MC2R expression accompanied by an ameliorated CT production (41), suggesting that ACTH-independent mechanisms underlying GC production may require a more prolonged stimulus. In this regard, MC2R internalization seems to be triggered by a prolonged ACTH binding to MC2R followed by an increase in p-PKA (42, 43). Moreover, *in vitro* evidence showed that, at least, MC2R desensitization results from a regulatory mechanism implicating MC2R internalization by clathrin-mediated endocytosis (42, 44, 45), which also appears to be insensitive to PKA activation from heterologous sources other than ACTH (44). Under non-stressful conditions, nearly 28% of internalized MC2R may be recycling to the cell surface, while the remaining fraction may be subjected to lysosomal degradation (43). Since MCR2 immunoreactivity after 14 days pi seems to be mostly localized within cell cytoplasm, it is conceivable that under prolonged stressful conditions like Tc infection, mechanisms about MC2R protein internalization and degradation are boosted, reinforcing MCR2 desensitization. Moreover, transcriptional activity of the Mc2r gene may be upregulated by diverse transcription factors, like JDP2 (Jun dimerization protein 2) (46), FOXL2 (Forkhead box protein L2), or NR5A1 (steroidogenic factor 1) (47), which, in the context of Tc infection, may be disturbed. Further studies are needed to address such issue.

The ACTH-GC dissociation taking place in the late phase of infection may be explained by PGE2 stimulation of fasciculate cells. PGE2 is produced in response to inflammation, injury or mechanical stress and may also stimulate steroid production partly by triggering adrenal cAMP production (48). PGE2 synthase is the enzyme responsible for the PGE2 synthesis, being likely that autocrine PGE2-stimulated expression of the PGE2 receptor (22, 49, 50) was exerting a positive regulatory role on GC synthesis. Strikingly, during Tc infection, PGE2 synthase showed a noticeable biphasic response, compatible with both ACTH-dependent and -independent phases, suggesting that autocrine PGE2 production may favor CT secretion. In the early phase, PGE2 may stimulate CT release from adrenal cells synergistically with ACTH, increasing cAMP. On the other hand, during the ACTH-hyporesponsive period, CT secretion may be sustained by the PGE2 produced because of increased PGE2 synthase bioavailability, as shown in Figure 4C. Furthermore, induction of cAMP by PGE2 when the PKA signaling cascade was shutting off may favor EPAC-mediated actions (51, 52). The abundance of EPAC2 protein in adrenal glands from infected mice after 14 days pi, along with its increased mRNA the day before, is highly suggestive that cAMP-activated EPAC, rather than PKA, mediates GC production during the late phase of infection.

Studies in human adrenal adenomas led to propose the existence of an alternative pathway of GC synthesis governed by IL-1β and IL-1RI, instead of ACTH (53). Indeed, similar mechanisms have been proposed for IL-1α (38). In our model, both systemic and intra-adrenal IL-1β may elicit its effects by promoting the local secretion of PGE2 or other factors that stimulate the steroidogenic machinery in both ACTH-dependent and independent phases. However, IL-1β/IL-1RI signaling seemed to be slightly depressed in the second phase; in this sense, IL-1β does not seem to contribute to the mechanisms sustaining the ACTH-independent GC production. While IL-1β was found to promote catecholamine production by adrenomedullar cells (54, 55), a synergy between IL-1β and catecholamines in driving GC secretion in this experimental model sounds unlikely. Our previous studies in the late phase of experimental Chagas disease in C57BL/6 female mice indicated that neither infection nor sympathectomy affected noradrenaline contents in adrenal glands (56).

Collectively, our data strongly point to the existence of two phases dealing with GC synthesis during Tc infection in mice, an initial phase that matches with the well-known ACTH-dependent pathway, followed by a second one characterized by an ACTH-hyporesponsive state. The inflamed adrenal microenvironment may also tune the production of intracellular mediators influencing GC synthesis like PGE2 synthase and EPAC2, which emerge as driving forces for GC production during progressive Tc infection. Lastly, CT production seems to be associated to a biphasic action of PGE2, implying that the effect of PGE2/cAMP in the ACTH-independent phase may be mediated by EPAC2.

Increasing amount of the experimental evidence indicated that the degree of dissociation between ACTH and GC secretion is of clinical relevance, as it has been associated with the level of complications of sepsis, surgery, malignant disease, and depression. In the context of human Chagas disease, beyond the disturbed HPA response in terms of the CG/dehydroepiandrosterone ratio, GC levels fell within normal levels (57, 58). Nevertheless, it is possible that during the acute symptomatic phase of human Chagas disease, as seen in the highly lethal oral acute infection (28, 29), the regulation of GC production may be like the one seen in the experimental model. Further studies are needed to address whether oral Chagas disease outcomes are linked to ACTH-GC decoupling response.

## Data Availability Statement

The raw data supporting the conclusions of this article will be made available by the authors, without undue reservation, to any qualified researcher.

## Ethics Statement

All animal procedures were performed according to the Guide for the Care and Use of Laboratory Animals (NIH), and approved by Institutional Committees (Bioethics, Animal Care and Use, and Biosecurity Committees, FCM-UNR; Resolutions No. 3486/2013 and 4976/2013).

## Author Contributions

VC, AP, and SV conceived and designed the experiments and contributed reagents, materials, analysis tools. ES, ER, FG, RF, AP, and SV performed the experiments. ES, RF, AP, and SV analyzed the data. AP, OB, and SV interpreted the data and wrote the paper.

### Conflict of Interest

The authors declare that the research was conducted in the absence of any commercial or financial relationships that could be construed as a potential conflict of interest.
