# Supplementary material for: Evidence in Favor of an Alternative Glucocorticoid Synthesis Pathway During Acute Experimental Chagas Disease
Source: Front Endocrinol (Lausanne). 2020 Jan 8;10:866. doi: 10.3389/fendo.2019.00866 (PMC6961479; doi:10.3389/fendo.2019.00866)
Supplement: Supplementary file 1 [file Image_1.pdf]

Supplementary Figure 1, da Silva et al

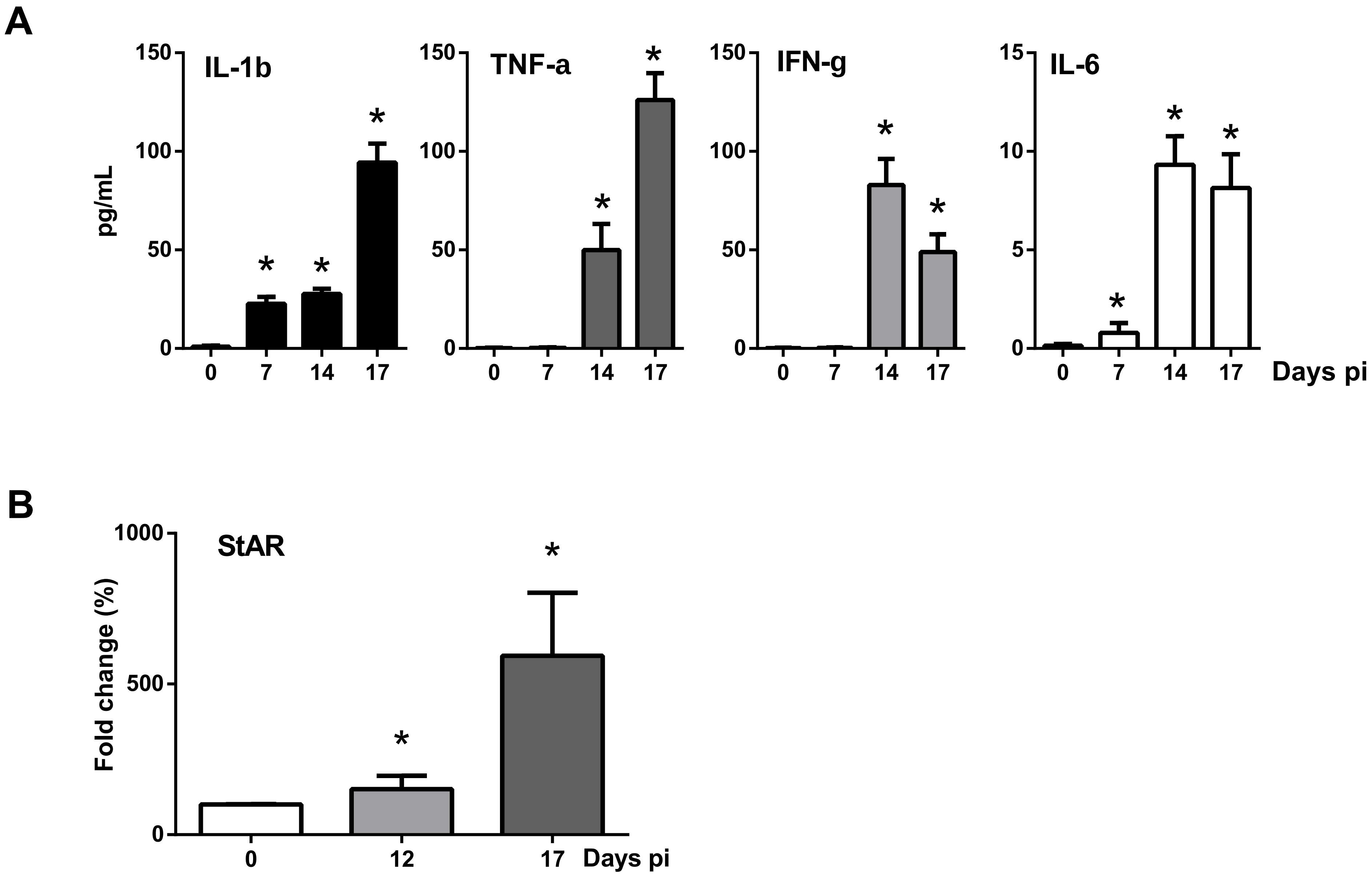

LEGEND SUPPLEMENTARY FIGURE

Supplementary Figure 1: A. Plasma levels of HPA axis-activating cytokines during T. cruzi infection. Plasma levels of IL-1β, TNF-α, IFN-γ and IL-6 were assessed throughout acute infection. **B.** StAR ARNm expression in adrenal glands from Tc-infected mice during infection, assayed by RT-qPCR. Data are expressed as mean ± SEM, from 3-5 mice/group/day. Results are representative from at least three independent experimental rounds. In all cases *$p<0.05$ vs. day 0 pi. n.d.: Non-detectable; pi: post-infection; AU: arbitrary units.
